# Magnetic Resonance Imaging-Based Robotic Radiosurgery of Arteriovenous Malformations

**DOI:** 10.3389/fonc.2020.608750

**Published:** 2021-03-09

**Authors:** Tobias Greve, Felix Ehret, Theresa Hofmann, Jun Thorsteinsdottir, Franziska Dorn, Viktor Švigelj, Anita Resman-Gašperšič, Joerg-Christian Tonn, Christian Schichor, Alexander Muacevic

**Affiliations:** ^1^Department of Neurosurgery, University Hospital, LMU Munich, Munich, Germany; ^2^European Cyberknife Center Munich-Grosshadern, Munich, Germany; ^3^Institute of Neuroradiology, University Hospital, LMU Munich, Munich, Germany; ^4^Division of Neurology, University Medical Center Ljubljana, Ljubljana, Slovenia

**Keywords:** CyberKnife, radiosurgery, stereotactic, arteriovenous malformation, Gammaknife surgery

## Abstract

**Objective:**

CyberKnife offers CT- and MRI-based treatment planning without the need for stereotactically acquired DSA. The literature on CyberKnife treatment of cerebral AVMs is sparse. Here, a large series focusing on cerebral AVMs treated by the frameless CyberKnife stereotactic radiosurgery (SRS) system was analyzed.

**Methods:**

In this retrospective study, patients with cerebral AVMs treated by CyberKnife SRS between 2005 and 2019 were included. Planning was MRI- and CT-based. Conventional DSA was not coregistered to the MRI and CT scans used for treatment planning and was only used as an adjunct. Obliteration dynamics and clinical outcome were analyzed.

**Results:**

215 patients were included. 53.0% received SRS as first treatment; the rest underwent previous surgery, embolization, SRS, or a combination. Most AVMs were classified as Spetzler-Martin grade I to III (54.9%). Hemorrhage before treatment occurred in 46.0%. Patients suffered from headache (28.8%), and seizures (14.0%) in the majority of cases. The median SRS dose was 18 Gy and the median target volume was 2.4 cm³. New neurological deficits occurred in 5.1% after SRS, with all but one patient recovering. The yearly post-SRS hemorrhage incidence was 1.3%. In 152 patients who were followed-up for at least three years, 47.4% showed complete AVM obliteration within this period. Cox regression analysis revealed Spetzler-Martin grade (P = 0.006) to be the only independent predictor of complete obliteration.

**Conclusions:**

Although data on radiotherapy of AVMs is available, this is one of the largest series, focusing exclusively on CyberKnife treatment. Safety and efficacy compared favorably to frame-based systems. Non-invasive treatment planning, with a frameless SRS robotic system might provide higher patient comfort, a less invasive treatment option, and lower radiation exposure.

## Introduction

Cerebral arteriovenous malformations (AVMs) consist of a complex tangle of abnormal blood vessels - the nidus, which does not clearly correspond to an artery or vein and lacks a physiological capillary bed. With an annual detection rate of one per 100,000, AVMs are a rare but significant vascular pathology (1). If ruptured, AVMs can cause substantial morbidity and mortality. Current treatment protocols are based on a detailed assessment of the risk of spontaneous bleeding during the natural course of the disease versus the risk of invasive AVM treatments (2).

Stereotactic radiosurgery (SRS), alone or in combination with embolization, is an important treatment option for intracranial AVMs, especially if the lesion is not eligible for surgery or embolization or if only partial occlusion can be achieved after embolization (3). In general, a high prescription dose between 15 and 25 Gy is required to obliterate AVMs by SRS. However, a prolonged median time to complete obliteration of around three years is described in the literature and is a known limitation of radiosurgery (4–6). Determinants of obliteration latency have been investigated in the past for various SRS systems such as Gamma Knife (7) and LINAC systems (8) but studies focusing on CyberKnife treatment of AVMs is sparse.

The comprehensive diagnostic work-up of AVMs is based on magnetic resonance imaging (MRI) for topography and digital subtraction angiography (DSA) for flow dynamics. Additionally, a planning CT angiography is necessary for image coregistration in all above-mentioned SRS systems. The Gamma Knife system is frame-based (9) and LINAC SRS systems are usually (but not exclusively) frame-based as well (10), meaning that a stereotactic frame has to be mounted to the patient before they receive the planning CT which is later referenced to the MRI. If the practitioner needs an exact overlay of the DSA with the MRI and CT images for nidus definition, the DSA has to be acquired with a stereotactic frame as well (11, 12). The acquisition of a stereotactic DSA is a time-consuming procedure compared to conventional DSA because the frame has to be mounted using local anesthesia and many patients even require general anesthesia throughout the whole acquisition process. DSA with external localizers or fiducials was shown to be feasible but is not yet established in clinical routine (13–15).

In contrast to frame-based systems, the CyberKnife system (Accuray, Inc., Sunnyvale, CA) relies on real-time image correction during the procedure without the necessity of a stereotactic frame (16). Therefore, computerized treatment planning is solely based on CT and MRI data. DSA is usually used solely as adjunct information without exact image-overlay.

Indeed, MR angiography (MRA) was shown to provide the possibility of non-invasive AVM examination without the need for an additional invasive DSA (17–23). The integration of high-resolution MRI scans into the treatment planning process of the CyberKnife and their use in subsequent follow-up studies have been proven feasible (24).

The objective of this study was to analyze the efficacy and safety of CyberKnife SRS treatment of intracranial AVMs in a large cohort of patients. Planning was based on coregistered MRI and CT images only, using a conventional non-coregistered DSA solely as an adjunct.

## Methods

### Study Design

**Figure 1:**
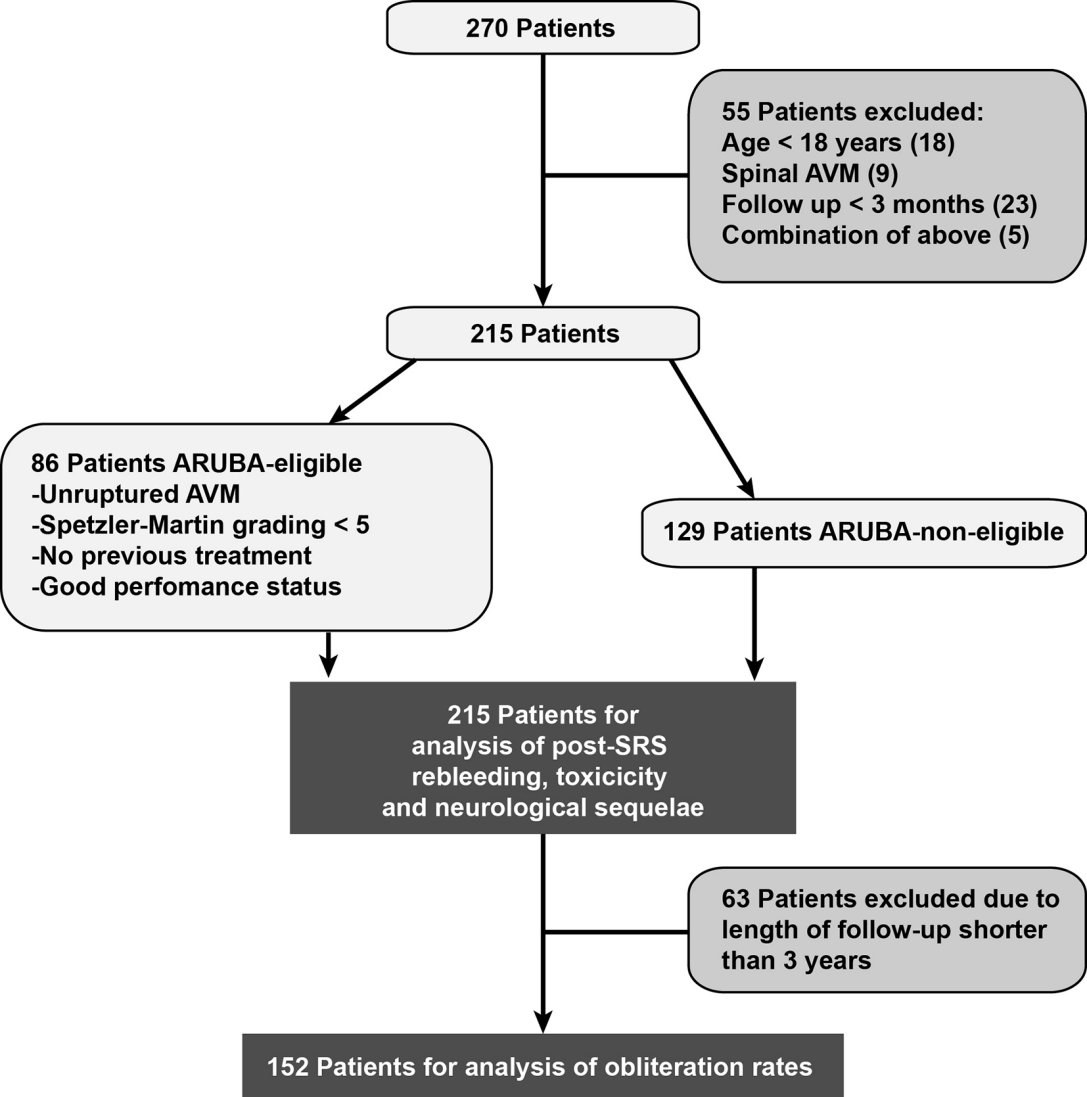
Patient inclusion scheme.

In this retrospective, single-center, non-randomized study patient characteristics, pre-treatment status, radiation parameters, and outcome were collected in our database. Between 2005 and 2019, 270 patients received CyberKnife SRS for cerebral AVMs and were screened for eligibility for this study. Patients were excluded if they were below the age of 18 (n = 18), were treated for spinal AVM (n = 9), or if the follow-up period was less than 5 months (n = 23). Five additional patients were excluded due to a combination of those criteria. Accordingly, 215 patients were included in this retrospective analysis (**Figure 1**). Subgroup analysis was performed in patients meeting the inclusion criteria of the ARUBA study (“A Randomized Trial of Unruptured Brain AVMs”): unruptured AVMs, Spetzler-Martin grade < V, no previous treatment and good Karnofsky performance status ≥ 80% before treatment (25). Obliteration rates were evaluated in patients with at least three years of follow-up.

Written consent to use the collected data for this retrospective analysis was obtained from every patient before treatment. All procedures were in accordance with institutional guidelines. This study was approved by the institutional review board (accession number 20-250KB).

### Study Parameters

**Figure 2:**
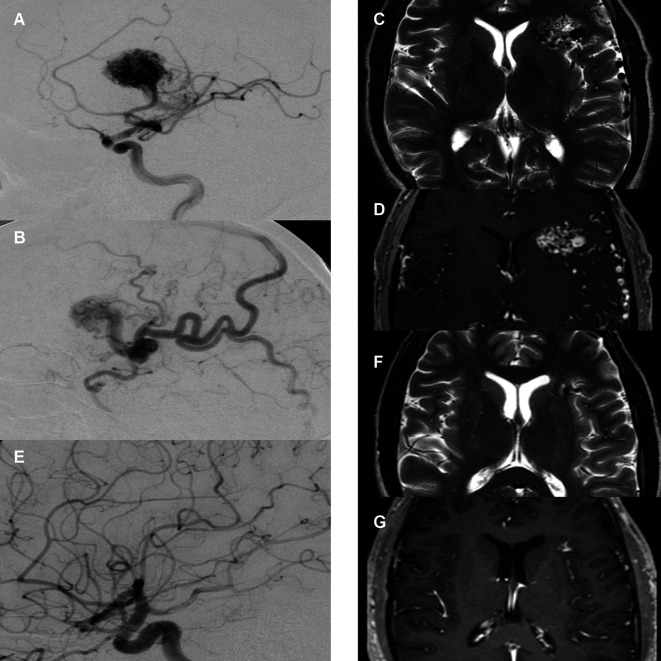
Case illustration. **(A)** Pre-treatment digital subtraction angiography (DSA), lateral view, arterial phase. Depiction of right frontal arteriovenous malformation (AVM) supplied by the medial cerebral artery with large nidus. **(B)** Pre-treatment DSA, lateral view, early venous phase. Depiction of diluted cortical veins. **(C)** Pre-treatment T2-sequence magnetic resonance imaging (MRI) with depiction of AVM nidus and diluted veins. **(D)** Pre-treatment T1-MR-angiography of the same area. **(E)** 2-year post-treatment follow-up DSA, arterial phase with no residual nidus or early venous drainage. **(F)** 2-year post-treatment follow-up T2-sequence MRI with depiction of a small residual lesion without T2-hyperintense radiation induced changes. **(G)** 2-year post-treatment follow-up T1-MR-angiography with depiction of small residual contrast enhancement.

Basic demographics and AVM specifications were extracted from our database. Pre-treatment work-up consisted of a DSA (**Figures 2A, B**), a CT angiography (with contrast injection) as well as a dedicated MRI study with 1 mm slice thickness (**Figures 2C, D**). The pre-treatment non-stereotactic (acquired without stereotactic frame) DSA solely served as adjunct information and was not coregistered with treatment planning CT and MRI studies. AVMs were classified using the Spetzler-Martin grading system (26), with a score of VI being attributed to inoperable lesions. Furthermore, the radiosurgery-based AVM score was calculated (27). It is a score on a continuous scale which includes AVM volume, patient age and deep localization and was previously shown to predict outcome after radiosurgery (28, 29).

### CyberKnife Treatment

**Figure 3:**
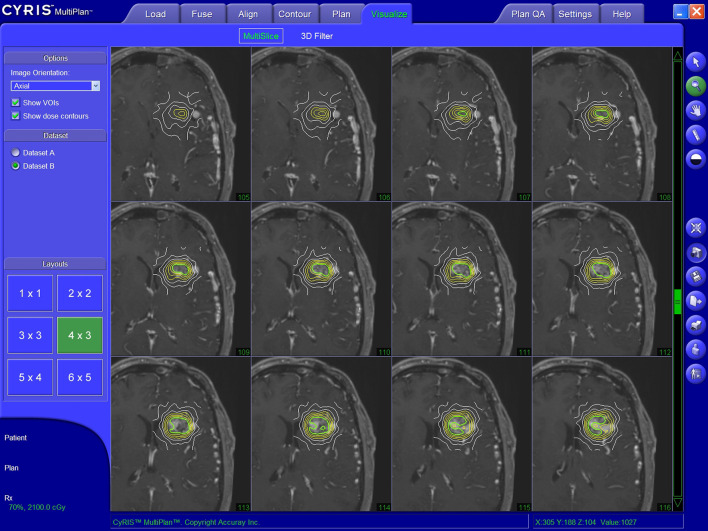
Illustratory target volume planning achieved with the MultiPlan planning software (Accuray Inc., Sunnyvale, CA, USA).

The CyberKnife robotic SRS system consists of a 6-MV compact linear accelerator mounted on a computer-controlled, 6-axis robotic manipulator (16). Integral to the system are orthogonally positioned x-ray cameras for image acquisition during treatment. These images are processed automatically to identify specific cranial bone structures. The information is then referenced to the CT angiography study to determine the exact position of the SRS target in real-time and to compensate for changes in patient position during treatment. The treatment principle of the CyberKnife represents a noncoplanar, nonisocentric dose delivery. The precision of the CyberKnife technology was shown to be comparable to published frame-based SRS systems (30). Dose determination and target volume planning was achieved with various versions of the MultiPlan and Precision planning softwares (Accuray Inc., Sunnyvale, CA, USA) analogously to previous publications (24) (**Figure 3**).

For each patient, a 1 mm isotropic T1 post-gadolinium and a 1 mm isotropic T2 MRI sequence were coregistered with the CT images to verify correct AVM topography during dose planning. Although primary DSA imaging was taken into account during target delineation, it was not coregistered with the other imaging modalities. Volume-staged CyberKnife SRS (subdivision of the target volume with sequential CyberKnife SRS sessions separated by intervals of days to weeks) was not performed. In patients with multiple target volumes, the absolute target volume and dose were used for further analysis.

### Definition of Obliteration and Follow-Up

After SRS, patients were followed up clinically and by MRI scans at 6-month intervals. The standard MRI protocol included 1 mm isotropic T1 post-gadolinium and 1 mm isotropic T2 morphological sequences and a 3D TOF MRA. Volumetric characterization of the nidus was performed with various versions of the MultiPlan and Precision planning software (Accuray Inc., Sunnyvale, CA, USA).

In line with existing literature (31), partial obliteration in MRI was defined as a reduction of the original AVM nidus volume of 50-95%. Complete obliteration was defined as > 95% reduction of the original AVM nidus volume combined with absence of early contrast filling of a draining vein in time-resolved MRA (**Figures 2F, G**).

If the MRI scan indicated complete obliteration, the patient was recommended to obtain a DSA to verify complete AVM obliteration. If the MRI did not indicate a complete obliteration, a DSA was recommended after three years at the latest. Although DSA was recommended to all patients, only some of the patients had this test performed. However, both patients with and without follow up DSA were included in the analysis.

In DSA follow-up imaging, partial obliteration was defined as disappearance of the AVM nidus with persistence of an early filling draining vein, indicating that residual shunting is still present. Complete obliteration was defined as disappearance of the AVM nidus without any early filling draining vein (**Figure 2E**).

### Statistics

An univariate analysis was performed for factors favoring AVM obliteration within three years. For this purpose, continuous variables were tested for normal distribution using the Shapiro-Wilk test, with only age being found to be normally distributed. Consequently, the descriptive statistics in the tables are listed as median and interquartile range (IQR). IQR measures statistical dispersion in non-normally distributed data, equal to the difference between 75th and 25th percentiles, or between upper and lower quartiles. The t-test was used to compare age and the Mann-Whitney U-test to test all other continuous variables between patients with and without complete obliteration within 36 months. The distribution of ordinal and nominal scaled variables between patients with and without complete obliteration within three years was analyzed using the exact Fisher test and the Chi-square test. The cumulative probability of partial and complete obliteration was evaluated using Kaplan-Meier statistics. Factors associated with obliteration within three years were tested in a multivariate Cox regression model. A significance level of p < 0.05 was chosen for the tests. SPSS version 25 (IBM) was used for statistical calculations.

## Results

### Patient Demographics

Of 215 included patients, 49.3% were female, and the median age was 40.4 years (range 18.3–79.8 years). The overall rate of cardiovascular risk factors was low, with arterial hypertension being the main contributor (n = 18). One hundred seventy-four (80.9%) patients had a supratentorial localization of the AVM, while 41 (19.1%) patients presented with infratentorial localization. The majority consisted of Spetzler-Martin grade I to III AVMs (118, 54.9%), while 69 (32.1%) lesions were classified as Spetzler-Martin grade IV or V and 28 (13.0%) were inoperable AVM lesions, classified as Spetzler-Martin grade ≥ IV.

The most frequent presenting symptom was headache (n = 62, 28.8%). AVM associated hemorrhage before treatment was detected in 99 (46.0%) patients. In 15 cases, hemorrhaged AVMs were operated upon to evacuate the hematoma. In 39 cases, the bleeding was eloquently located and did not justify the risk of surgical decompression. In the other 45 cases of the 99 with an associated hemorrhage, the bleeding was small, asymptomatic and did not require hematoma evacuation. Of all patients with an associated hemorrhage, 16 (7.4%) were asymptomatic, 41 (19.1%) had neurological deficits, 13 (6.0%) had headache, 6 (2.8%) presented with epilepsy and 23 (10.7%) had a combination of those symptoms (**Table 1**).

**Table 1:** Patient demographics and characterization of arteriovenous malformations.

Variable		Value
**Subjects, N**		215
**Age, median years ± SD**		40.4 ± 13.3
**Sex, female**		106 (49.3%)
**Hypertension**		18 (8.4%)
**Nicotine abuse**		8 (3.7%)
**AVM side, left**		116 (54.0%)
**AVM size**	Small (diameter < 3 cm) Medium (diameter 3–6 cm) Large (diameter > 6 cm) Maximum volume	121 (56.3%) 49 (22.8%) 45 (20.9%) 35.7 cm³
**AVM localization**	Lobar Infratentorial Eloquent	148 (68.8%) 41 (19.1%) 72 (33.5%)
**AVM venous drainage pattern**	Superficial only Any deep	136 (63.3%) 79 (36.7%)
**Spetzler-Martin grade**	I II III IV V VI	13 (6.0%) 42 (19.5%) 63 (29.3%) 48 (22.3%) 21 (9.8%) 28 (13.0%)
**Radiosurgery-based AVM score**	Median (IQR) Range	1.36 [1.11–1.70] 0.45–4.67
**AVM-associated arterial aneurysm**		10 (4.7%)
**AVM-associated hemorrhage**		99 (46.0%)
**Previous treatment**	None Embolization Surgery Surgery & embolization SRS SRS & embolization SRS & surgery & embolization	114 (53%) 62 (28.8%) 10 (4.7%) 12 (5.6%) 6 (2.8%) 10 (4.7%) 1 (0.5%)
**Clinical presentation**	Seizure Headache Focal deficit Other deficit Asymptomatic	30 (14.0%) 62 (28.8%) 63 (29.3%) 36 (16.7%) 35 (16.3%)

If not otherwise indicated, frequencies are presented as n (%). AVM, arteriovenous malformation; SD, standard deviation; IQR, interquartile range; SRS, stereotactic radiosurgery; Eloquent is any AVM location involving the sensorimotor, language, or visual cortex; the hypothalamus and thalamus; the internal capsule; the brainstem; the cerebellar peduncles; or the deep cerebellar nuclei. Associated arterial aneurysms are flow-related aneurysms located on a feeding artery or within the AVM nidus (so-called intranidal aneurysms). Other deficits include gait ataxia, vertigo, cognitive deficits, and fatigue.

While 114 (53.0%) patients received no previous treatment, 23 (10.7%) were previously subjected to surgery alone or in combination with embolization, 16 (7.4%) were previously subjected to SRS, alone or in combination with embolization, and 62 (28.8%) were previously subjected to embolization alone.

Of the 23 patients who received a previous surgery, 11 were internal referrals and 12 were externally referred to receive CyberKnife SRS at our center. Of the 11 internal referrals, 5 only received surgical hematoma evacuation, because the AVM was non-amenable to resection. Five showed minimal residual AV-shunting on postoperative DSA and one patient had a partial AVM resection.

### Treatment Details and Follow-Up Imaging

Of all AVMs treated with CyberKnife SRS, 210 (97.7%) had single targets at the nidus while five (2.3%) had multiple target volumes. The median dose was 18 Gy (range, 15–30 Gy). Of all patients treated for AVMs with CyberKnife, all but one patient received a maximum of 25 Gy. There was one patient who received 29.58 Gy. He had a very small high flow AVM/fistula with a volume of 0.4 cc which explains the high focal dose. The median prescription isodose line was 85%. There was a wide range of target volumes (0.1 cm³ to 35.7 cm³) with the median being 2.4 cm³. The Spearman-Rho correlation coefficient between target volume and coverage was 0.157. This value shows that smaller tumors do not manifest an increased coverage or vice versa. The range of follow-up was 5.6 to 165.9 months, with a median value of 40.2 months. All patients were recommended to obtain a DSA after 3 years or if obliteration was suspected in MRI. In all 152 patients who were included in the efficacy analysis (minimum follow-up of three years), 76 (50.0%) were followed-up by DSA while the rest failed to provide a DSA follow-up study, mostly due to its invasive nature and logistic effort (**Table 2**).

**Table 2:** CyberKnife radiosurgery and follow-up imaging.

Variable		Value
**Dose, median Gy (IQR)**		18.0 [17.0– 20.0]
**Prescription isodose, median % (IQR)**		85.0 [70.0–85.0]
**Target volume**	Median cm³ (IQR) Range cm³	2.4 [0.9–5.0] 0.1–35.7
**Homogeneity index**	Median (IQR)	1.18 [1.18–1.43]
**Conformity index**	Median (IQR)	1.18 [1.12–1.25]
**Coverage to GTV**	Median (IQR)	96% [92.5–97.7%]
**Follow-up period**	Median months (IQR) Range months	40.2 [21.6–786] 5.6–165.9
**Follow-up imaging in all patients**	MRI MRI and DSA	139 (64.7%) 76 (35.3%)
**Follow-up imaging in patients included in efficacy analysis**	MRI MRI and DSA	76 (50.0%) 76 (50.0%)
**Discrepancies between MRI and DSA**	MRI inconclusive, DSA shows complete obliteration MRI suggests higher grade of obliteration than DSA	10 (5.2%) 0
**Post-SRS hemorrhage**	Overall With pre-SRS hemorrhage (N 93) ARUBA-eligible (N 86)	12 (5.6%) 6 (6.5%) 0
**Yearly post-SRS hemorrhage risk**	Incidence (95% CI)	1.3% [0.7–2.3%]

When not otherwise indicated, frequencies are presented as n (%). SRS, stereotactic radiosurgery; MRI, magnetic resonance imaging; DSA, digital subtraction angiography; IQR, interquartile range; CI, confidence interval; GTV, gross treatment volume.

### Neurological Deficits and Treatment-Related Morbidity

Patients presented with a median Karnofsky performance status of 90% (range, 40%–100%) before SRS. The median Karnofsky performance status did not change at first and last follow-up.

Thirty patients (14.0%) presented with symptomatic epilepsy before SRS treatment. After SRS treatment, 29 patients (13.5%) developed new seizures. Of all 59 patients manifesting symptomatic epilepsy before or after SRS, 36 (16.7%) were adequately controlled with medication.

During the follow-up period, 73 (73.7%) of 99 patients with neurological deficits recovered completely or partially.

There were 11 (5.1%) new neurological deficits after SRS, with ten recovering partially or completely (hemiparesis, cerebellar symptoms, aphasia, cognitive deficits, fatigue) and one visual field deficit not recovering completely. Bivariate analysis revealed that the proportion of patients with new deficits after SRS was higher in those that received previous SRS (17.6% versus 4%, p = 0.015). Similarly, AVMs with a high Spetzler-Martin grading were significantly more at risk for a new deficit after SRS. While patients with a Spetzler-Martin grade I or II lesion developed no new deficit, patients with grade III or IV lesions developed three (2.7%) new deficits and patients with grade V or VI lesions developed eight (16.3%) new deficits (p < 0.001). Furthermore, a lower median prescription isodose line was associated with new deficits (70% versus 85%, p = 0.022).

Twelve (5.6%) patients developed an AVM related intracerebral hemorrhage after SRS, two of whom died and seven of whom presented with a new neurological deficit. A bivariate risk factor analysis showed that higher single dose (22.5 Gy versus 17.5 Gy, p = 0.003) and a lower median prescription isodose line (67.5% versus 85%, p < 0.001) were associated to hemorrhage after SRS.

The yearly post-SRS hemorrhage incidence was 1.3% in patients with no or partial obliteration. Four additional deaths were non-related to the AVM or SRS treatment (**Table 3**).

**Table 3:** Morbidity and mortality.

Variable		Value
**Karnofsky performance status before SRS**	Median (IQR) Range	90% [90–100%] 40–100%
**Karnofsky performance status at first follow-up**	Median (IQR) Range	90% [90–100%] 40–100%
**Karnofsky performance status at last follow-up**	Median (IQR) Range	90% [90–100%] 40–100%
**Seizures**	None Presenting symptom Onset after radiosurgery	156 (72.3%) 30 (14.0%) 29 (13.5%)
**Headache**	None Presenting symptom Onset after radiosurgery	147 (68.4%) 62 (28.8%) 6 (2.8%)
**Neurological deficits before SRS**	None Visual field deficits Monoparesis Hemisyndrome without aphasia Vertigo Cerebellar symptoms Hypesthesia Diplopia Aphasia Fine motor skills Facial palsy Hemisyndrome with aphasia Cognitive deficits Fatigue	116 (54%) 25 (11.6%) 12 (5.6%) 10 (4.7%) 9 (4.2%) 10 (4.7%) 7 (3.3%) 7 (3.3%) 5 (2.3%) 5 (2.3%) 3 (1.4%) 3 (1.4%) 2 (0.9%) 1 (0.5%)
**Course of neurological deficits (N 90) after SRS**	No recovery Partial recovery Full recovery Worsened after SRS, no recovery Worsened after SRS, partial recovery	24 (26.7%) 40 (44.4%) 18 (20.0%) 2 (2.2%) 10 (11.1%)
**New deficits after SRS**	Overall Facial palsy (full recovery) Monoparesis (partial recovery) Coordination (full recovery) Visual field deficits (no & partial recovery) Aphasia (partial recovery) Hemisyndrome (partial recovery) Cognitive deficits (partial recovery) Fatigue (full recovery)	11 (5.1%) 1 1 2 3 1 1 1 1
**Death**	AVM related Non-related to AVM	2 (0.9%) 4 (1.9%)

When not otherwise indicated, frequencies are presented as n (%). AVM, arteriovenous malformation; IQR, interquartile range; SRS, stereotactic radiosurgery.

### Treatment Efficacy

Obliteration rates were calculated in 152 patients who were followed-up for at least 3 years. Of those, 72 (47.4%) had a complete AVM obliteration within the first 3 years after SRS and 80 (52.6%) had a persisting AVM lesion (**Table 4**). Of those without complete obliteration after three years, 31 (20.4%) eventually obliterated until last follow-up so that the cumulative complete obliteration rate was 67.7% (n = 103).

**Table 4:** Efficacy of CyberKnife radiosurgery.

Obliteration status 3 years after SRS in patients with ≥ 3 years follow-up (N 152)			Value
**No obliteration** **Partial obliteration** **Complete obliteration**			6 (3.9%) 74 (48.7%) 72 (47.4%)
**Univariate analysis**	**Complete obliteration within 3 years (N 72)**	**No complete obliteration within 3 years (N 80)**	**P-Value**
**Spetzler-Martin grade** **I** **II** **III** **IV** **V** **VI**	4 (5.6%) 21 (29.2%) 23 (31.9%) 10 (13.9%) 5 (6.9%) 9 (12.5%)	2 (2.5%) 10 (12.5%) 21 (26.3%) 18 (22.5%) 14 (17.5%) 15 (18.8%)	0.028
**Radiosurgery-based AVM score**	1.33 [1.02–1.63]	1.44 [1.19–1.86]	0.028
**Dose, Gy**	18 [17–21]	17 [16.5–19]	0.002
**Target volume, cm³**	1.44 [0.52–4.46]	3.69 [1.51–7.89]	< 0.001
**Cox-regression multivariate analysis**	**Odds ratio and 95% CI**		**P-Value**
**Spetzler-Martin grade**	2.21 [1.96–2.55]		0.006

When not otherwise indicated, frequencies are presented as n (%). Radiosurgery-based AVM score, dose and target volume are presented as median and interquartile range. AVM, arteriovenous malformation; SRS, stereotactic radiosurgery; MRI, magnetic resonance imaging; DSA, digital subtraction angiography; CI, confidence interval.

The median time to complete obliteration was 41.6 months and the median time to partial obliteration was 6.7 months.

**Figure 4:**
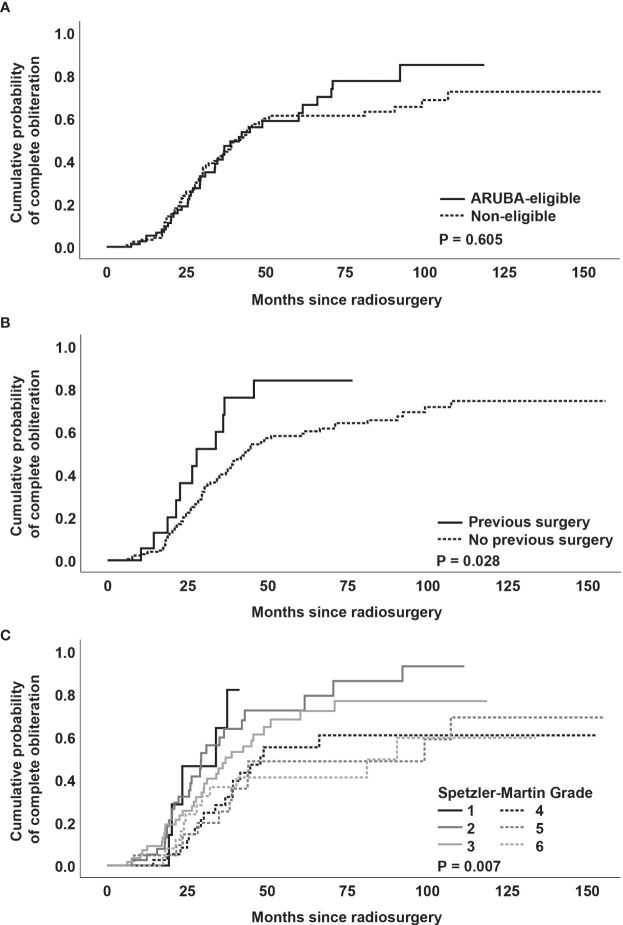
**(A)** Kaplan-Meier analysis of obliteration dynamics stratified by ARUBA-eligibility. **(B)** Kaplan-Meier analysis of obliteration dynamics stratified by previous surgery. **(C)** Kaplan-Meier analysis of obliteration dynamics stratified by Spetzler-Martin grade.

There was no significant difference between ARUBA-eligible (**Figure 1**) and ARUBA-non-eligible patients regarding median time to complete (41.6 months versus 42.1 months, P = 0.605) or partial obliteration (6.5 months versus 6.7 months, P = 0.078, **Figure 4A**).

Kaplan-Meier analysis revealed no significant difference in median time to complete obliteration for patients who received previous SRS versus patients who did not receive previous SRS (41.6 months versus 40.4 months, P = 0.166). Similarly, no difference was noted between patients who underwent neuroendovascular embolization before SRS versus patients who were not embolized before SRS (39.3 months versus 41.6 months, P = 0.604).

However, patients who received partial surgical resection of the AVM had a shorter median time to complete obliteration (27.8 months versus 43.0 months, P = 0.028, **Figure 4B**). In addition, obliteration dynamics significantly varied depending on the Spetzler-Martin grade (P = 0.007, **Figure 4C**). While complete obliteration after 3 years was achieved in 67% of patients with Spetzler-Martin grade I and II lesions, the obliteration rate for Spetzler-Martin grades III, IV, V, and VI was 52.3%, 35.7%, 26.3%, and 37.4%, respectively (P = 0.028).

A lower radiosurgery-based AVM score (P = 0.028), a smaller target volume (P < 0.001) and a higher prescription dose (P = 0.002) were also significantly associated with complete obliteration within 3 years in bivariate analysis.

When performing a multivariate Cox regression analysis with the above-mentioned significant variables from univariate analysis, only Spetzler-Martin grade (P = 0.006) was found as independent predictor of complete obliteration (**Table 4**).

## Discussion

We analyzed obliteration dynamics, bleeding events and complications in a large cohort of patients with ruptured and unruptured AVMs treated with the frameless CyberKnife SRS system.

After 3 years of follow-up, we found an overall complete obliteration rate of 47.4%. This obliteration rate is consistent with data on Gamma Knife and LINAC-based SRS, where obliteration rates between 30% and 58% were achieved (4, 32–36). The obliteration rate observed in our study must be placed in the context of an unfavorable patient selection with particularly difficult to treat AVMs, including a higher proportion of Spetzler-Martin grade IV to VI AVMs (45.1%) compared to other series (7.5%–22%) (4, 32–36).

Most larger studies on SRS treatment of cerebral AVMs were carried out on Gamma Knife and LINAC systems, while the literature on AVM treatment by CyberKnife is sparse. We found three studies with rather small sample sizes of less than 30 patients that had obliteration rates between 66% and 78% (37–39). One larger study evaluating the three-year outcome of 102 patients treated with CyberKnife SRS (40) found an obliteration rate of 71.5%, which was higher than ours. However, they mainly investigated the treatment efficacy of small AVMs (79% Spetzler-Martin grades I to II, 21% Spetzler-Martin grades III and IV) and did not consider obliteration dynamics, thereby attributing late complete obliteration the same importance as early obliteration. Late complete obliteration could be problematic due to the risk of dangerous rebleeding in the latency period (5).

Regarding safety, the annual hemorrhage incidence after SRS in our treatment cohort was low (1.3%). This is comparable with previously published literature on SRS with hemorrhage rates between 1.3% and 4.9% (4–6, 33, 36, 41). Of note, the yearly hemorrhage incidence was markedly lower compared to the medical arm of the ARUBA trial, where it was 2.2% (25).

During the follow-up period, 73.7% of neurological deficits before SRS either completely or partially resolved after treatment, which was comparable to other studies, which report a partial or full recovery rate around 70% (41).

New neurological deficits occurred in 11 (5.1%) patients, while seven of those were attributed to new hemorrhage. Similarly, two deaths after SRS were secondary to hemorrhage from the treated AVM. The rate of new neurological deficits was comparable to a large meta-analysis on SRS treatment of cerebral AVMs, where it was 8% (42).

Headache was found to be the most prevalent presenting symptom (28.8%) in our study and this proportion was similar to many other AVM studies (33, 36, 40, 42). While 14% of our cohort presented with symptomatic epilepsy before SRS, 13.5% additional patients developed new seizures after SRS. The rate of symptomatic epilepsy at presentation varies in the literature and ranges between 12% and 47% (25, 33, 36, 40–42). New onset of symptomatic epilepsy after SRS ranged between 3% and 10% in other studies (33, 43).

In multivariate analysis, only Spetzler-Martin grade remained an independent predictor of the obliteration status, as has equally been shown in other series (36). The fact that Spetzler-Martin grade is calculated based on AVM size, draining vein status and eloquence sufficiently explains why AVM size alone was not an independent predictor of obliteration.

While efficacy and safety of CyberKnife SRS of our data compared favorably to the literature, our study has several limitations. First, it was a retrospective study and therefore, no statistical power analysis was conducted in advance. Second, CyberKnife does not offer the possibility to coregister stereotactic DSA images with the CT or MRA, making it impossible to compare two patient cohorts with and without stereotactic DSA as a planning basis.

In addition, some patients refused to obtain DSA imaging during follow-up, mostly due to the invasiveness of the procedure, which may introduce a bias in the rate of complete obliteration. This is a common problem in clinical practice that similarly occurred in other large studies on SRS treatment of cerebral AVMs (32, 36, 40). To tackle this frequently observed limitation, one study with 136 patients analyzed the diagnostic accuracy of MRA regarding AVM obliteration after SRS. They showed a high sensitivity (85%) and specificity (95%) of MRA (31). In addition there is increasing evidence from small studies and case reports that newer time-resolved MRA sequences may be equal or even be better to assess AVM obliteration, when compared to DSA (44–47). While the scope of the present study was not to systematically compare the performance of MRA to DSA follow-up imaging, our obliteration and rebleeding rates were comparable to the literature (as discussed above) which speaks in favor of a correct assessment of obliteration, even in patients who were followed up by MRI only. However, we still advocate larger studies to systematically compare DSA with newer time-resolved MRA sequences in an effort to minimize radiation exposure for patients and potentially overcome the necessity of invasive DSA follow-up imaging in the future.

## Conclusion

Non-invasive treatment planning, based on MRI and CT angiography, with a frameless SRS robotic system is a safe and effective treatment option in otherwise difficult to treat intracranial AVMs.

Although data on radiotherapy of AVMs is available, this is—to the best of our knowledge—one of the largest series, focusing exclusively on CyberKnife treatment.

Obliteration dynamics and rebleeding rates compare favorably to conventional frame-based radiosurgery devices with stereotactic DSA-guided approaches and thereby might provide higher patient comfort, a less invasive treatment option and lower radiation exposure.

## Data Availability Statement

The raw data supporting the conclusions of this article will be made available by the authors upon reasonable request.

## Ethics Statement

The studies involving human participants were reviewed and approved by the ethics committee of the LMU Munich. The patients/participants provided their written informed consent to participate in this study.

## Author Contributions

AM, CS, and TG: conception and design of the study. FE, TH, TG, and AM acquired the data. TG analyzed the data and drafted the manuscript. FE, TH, JT, FD, CS, and AM critically revised the manuscript. All authors contributed to the article and approved the submitted version.

## Conflict of Interest

FD is a consultant for Balt, Phenox, and Cerus and received speaker honoraria from Cerenovus and Acandis.

The remaining authors declare that the research was conducted in the absence of any commercial or financial relationships that could be construed as a potential conflict of interest.
